# Exploring the Benefits of Ablation Grid Adaptation in 2D/3D Laser Ablation Inductively Coupled Plasma Mass Spectrometry Mapping through Geometrical Modeling

**DOI:** 10.1021/acs.analchem.3c00774

**Published:** 2023-06-01

**Authors:** Johannes T. van Elteren, Dino Metarapi, Kristina Mervič, Martin Šala

**Affiliations:** Department of Analytical Chemistry, National Institute of Chemistry, Hajdrihova 19, Ljubljana SI-1000, Slovenia

## Abstract

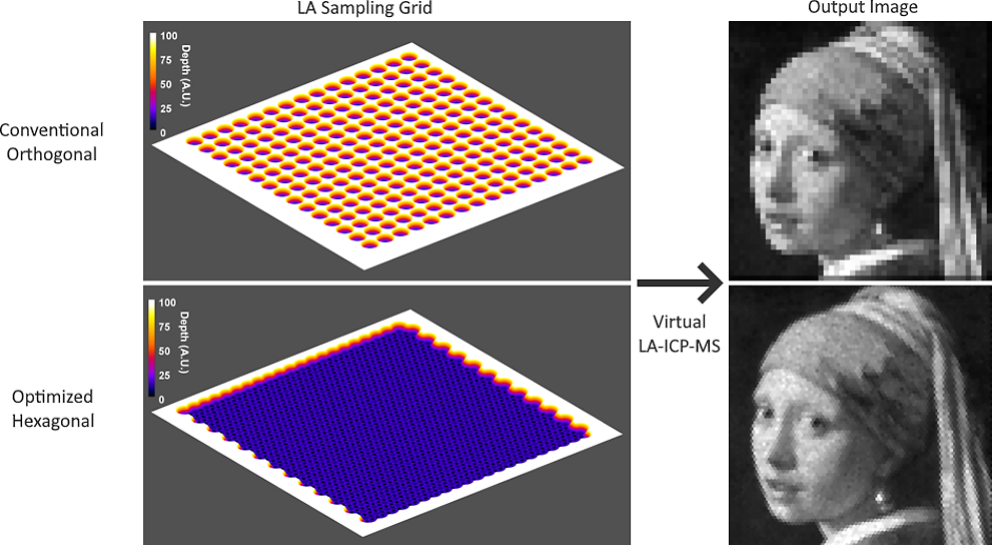

This study aims to investigate the potential benefits of adapting the ablating grid in two-dimensional (2D) and three-dimensional (3D) laser ablation inductively coupled plasma mass spectrometry in a single pulse mapping mode. The goals include enhancing the accuracy of surface sampling of element distributions, improving the control of depth-related sampling, smoothing the post-ablation surface for layer-by-layer sampling, and increasing the image quality. To emulate the capabilities of currently unavailable laser ablation stages, a computational approach using geometrical modeling was employed to compound square or round experimentally obtained 3D crater profiles on variable orthogonal or hexagonal ablation grids. These grids were optimized by minimizing surface roughness as a function of average ablation depth, followed by simulating the post-ablation surface and related image quality. An online application (https://laicpms-apps.ki.si/webapps/home/) is available for users to virtually experiment with contracting/expanding orthogonal and hexagonal ablation grids for generic 3D super-Gaussian laser crater profiles, allowing for exploration of the resulting post-ablation surface layer roughness and depth.

In order to reduce unwanted surface degradation during laser ablation of a material, analytical methods such as laser ablation inductively coupled plasma mass spectrometry (LA–ICP–MS) often utilize pulsed laser ablation devices with short pulse durations, ranging from picoseconds to nanoseconds. Additionally, generic Gaussian laser beam profiles are frequently shaped into flat-topped profiles to further decrease surface degradation caused by excessive heating. However, in practice, these beam profiles typically resemble a symmetric probability density function of high super-Gaussian order.^1−3^ Using spot-resolved surface ablation results in a more or less dimpled post-ablation surface,^4^ depending on the beam profile, ablation crater geometry, and the material’s absorption properties. Contracting the ablation grid in an orthogonal or hexagonal pattern can result in a smoother post-ablation surface and improved lateral and depth resolution.

Furthermore, layer-by-layer mapping may be improved as it requires overlapping ablation spots to counteract the non-top hat crater morphology. Riedo et al.^5^ decreased the pitch size to below the round spot size to generate smooth post-ablation surface layers in Sn/Ag solder bumps for repetitive scans up to 100 layers. To facilitate smoothing in consecutive layers, Van Malderen et al.^6^ and Westerhausen et al.^7^ laterally shifted the overlapping ablation spots in the different layers. The former publication presumed near Gaussian-type crater geometry produced in glass by a 3 μm round ablation spot, yielding a smooth post-ablation surface when scanning with a two-times overlap in 2D space.

When the ablation grid is contracted, the overlapping of ablation spots increases the laser spot density and results in more pixels being mapped in the same area when using single pulse LA–ICP–MS. This can lead to improved image quality, but if overlapping becomes excessive, it may cause blur.^8^ One of the potential benefits of hexagonal mapping is the fact that hexagonal pixels mimic the human visual system through hexagonal arrangement of sensory cells in the retina, fewer contour artifacts, better edge definition, higher pixel density, higher symmetry, etc.^9,10^ While current laser ablation stages are limited in their ability to implement hexagonal sampling, the benefits of using hexagons can be considered through simulations until hexagonal stages become available. The contraction approach is frequently applied in applications such as those encountered in industrial micro-machining,^11^ laser vision correction,^12^ dentistry,^13^ etc., to generate a high-quality surface finish. Most of these applications use laser devices with a Gaussian beam profile and optimized laser ablation conditions retrieved from geometrical modeling.^14−16^ Such a computational approach directly relates the area illuminated by the beam to the surface topography and induced roughness via spot-related 3D laser ablation crater geometries.^6,15,17,18^ The conditions computationally optimized for these non-analytical applications refer in general to grid geometry, spot geometry, feed speed, etc.

A similar geometrical modeling approach can be used to optimize ablation grids in LA–ICP–MS mapping by computationally compounding round and square ablation spots via 2D discrete convolution. By closer packing of ablation spots, a minimized surface roughness can be used as an indicator for optimal grid contraction. For surface sampling to a certain depth, contraction must be well-controlled to provide a smooth post-ablation surface for every depth layer. A general-purpose online application, not only for analytical lasers, demonstrates the effect of ablation grid contraction on the post-ablation surface layer roughness and depth for user-selected super-Gaussian crater profiles. Round and square ablation crater topographies associated with single laser ablation shots in a borosilicate glass matrix were obtained by optical profilometry and used for the optimization of the respective grids. Simulation illustrates how these optimized ablation grids can result in significantly reduced surface roughness in LA sampling, laying the foundation for more accurate, higher resolution 2D maps and smooth surfaces in 3D layer-by-layer mapping.

## Experimental Section

### Generation and Measurement of Individual LA Spots in Glass

A laser ablation system (193 nm ArF*; Analyte G2, Teledyne Photon Machines Inc., Bozeman, MT) equipped with a standard two-volume ablation cell (HelEx II, aerosol washout time of approximately 0.5 s) was used to produce 10 μm round and square ablation craters at a fluence of 4.08 J cm^–2^ and a helium carrier flow rate of 0.3 and 0.5 L/min for cup and cell, respectively. The morphologies of the craters were measured by optical profilometry (Zegage PRO HR, Zygo Corporation, CT) and were used as an input for the optimization of the orthogonal and hexagonal ablation grids through geometrical modeling. 3D information was recorded using a 50× magnification lens yielding a lateral resolution of 0.173 μm (equivalent to the step size) and surface topography repeatability better than 3.5 nm. Supporting Information-1 (videos in AVI format) shows the individual topographic laser ablation crater profiles of 50 registered 10 μm round and square spots which are composed of roughly 2600 and 3300 optical profilometry measurement points, respectively. Averaged crater profiles (Figure S2, Supporting Information, SI-2) were used for the optimization of the ablation grids by numerically compounding them on varying ablation grids until the simulated layer topography was as smooth as possible by minimizing the surface roughness. This geometrical modeling process can be performed by contracting the grids, either by equal reduction of the spot distance in both the *x*- and *y*-directions or through asymmetrical contraction. Additionally, we simulated how contraction of the grids can influence the image quality based on earlier developed computational protocols.^8,19,20^

### Geometrical Modeling Concept

**Figure 1:**
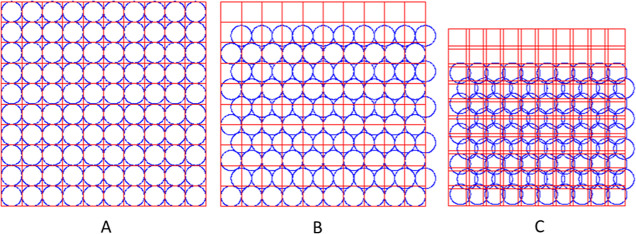
Ablation grids for round (blue) and square (red) ablation spots associated with conventional sampling (A), sampling based on closest packing on ideal orthogonal and hexagonal grids (B), and sampling on contracted grids for ablation spot profiles with an effective size smaller than the beam size (C). Simple mathematical calculations reveal that on the hexagonal grid in B, round spots can be packed 2/√3 = 1.155 times closer than square spots.

**Figure 2:**
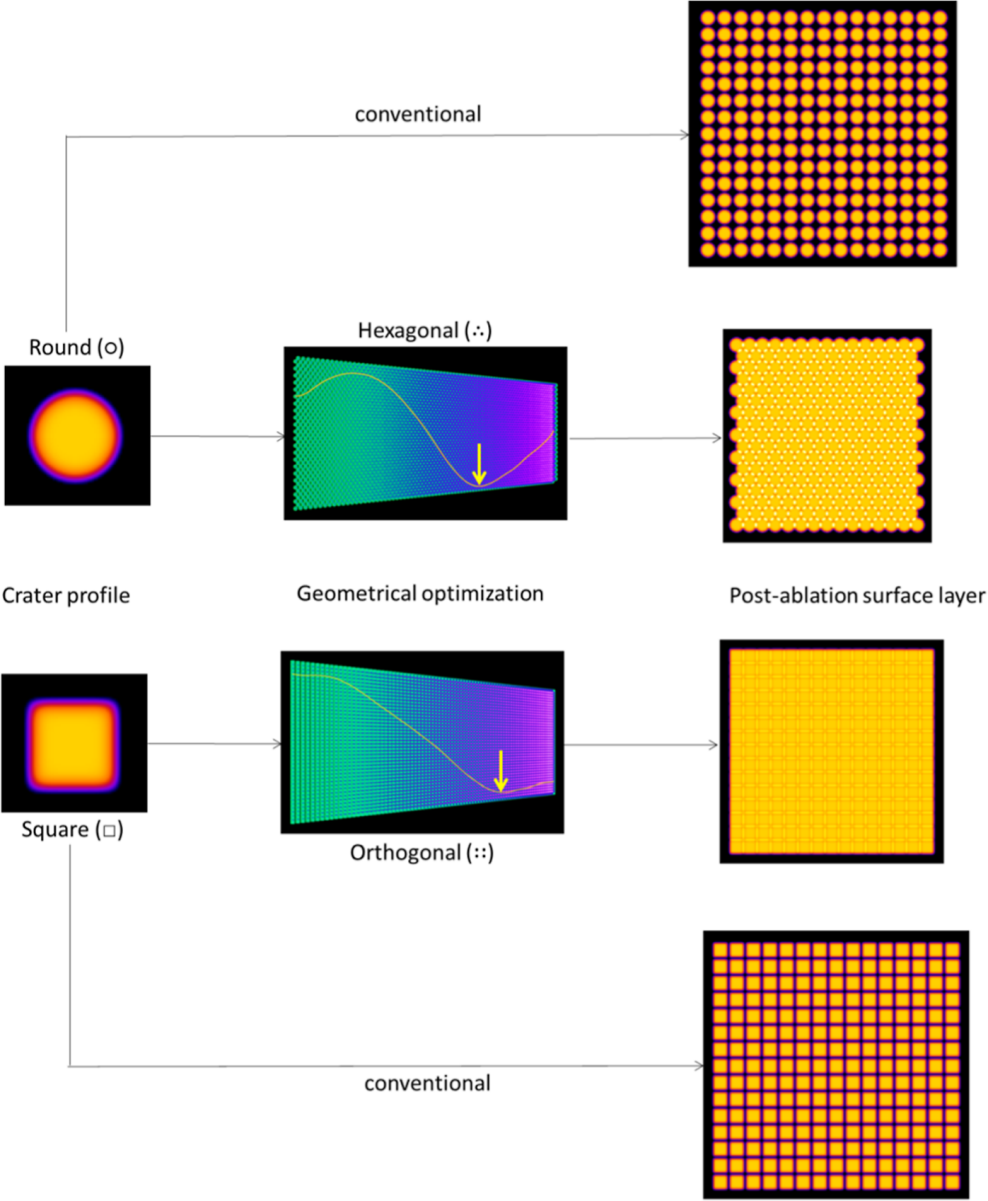
Surface sampling by laser beams associated with square (□) and round (○) ablation spots on conventional and geometrically optimized orthogonal (::) and hexagonal (∴) ablation grids, respectively. Through geometrical modeling, the surface roughness was minimized (see yellow arrows) by computationally contracting the ablation grids (from left to right). The post-ablation surface layers are all generated by 625 laser shots, indicating that by minimizing the surface roughness, the spot density increases significantly.

Conventional single pulse LA–ICP–MS mapping typically employs an orthogonal ablation grid based on the instrumental beam size, implying that round and square beam profiles with the same beam size have the same ablation grid and produce the same pixel size (Figure 1A). However, mathematically, ablation spots produced by true flat-topped square and round beam profiles, represented by perfect 3D cuboids and cylinders, can be most densely packed in an orthogonal and hexagonal arrangement, respectively (Figure 1B). However, if laser beam profiles are not truly flat-topped but show sloped edges, for densest packing of these spots, the ablation grid needs to be contracted as the effective spot size is smaller than the beam size (Figure 1C). Figure 2 illustrates how optimizing the LA orthogonal and hexagonal sampling grid significantly improves the post-ablation surface quality, with less “holes”, leading to a better representation of the element distribution in LA–ICP–MS mapping, and the possibility of scanning consecutive surface depth layers in a layer-by-layer fashion. This process of geometrical modeling is based on minimizing the surface roughness by computationally compounding ablation crater profiles through 2D discrete convolution on variable (contracting) ablation grids. The highest surface smoothness is used as an indicator for optimal ablation grid contraction. For optimizing the experimental ablation grids, the averaged crater topographies generated in a microscope glass slide (Figure S2, Supporting Information SI-2) were used.

### Software and Modeling

Numerical calculations and simulation modeling were performed using MatLab R2021b (MathWorks), and for data visualization, Origin software (Origin 2018, OriginLab Corporation, Northampton, MA) and ImageJ 1.4932^21^ were utilized. Several software routines were developed for specific purposes, including (i) generating theoretical 3D crater profiles based on super-Gaussian LA beam profiles, (ii) creating a database of registered, actual 3D crater profiles obtained through optical profilometry (see sifile1), (iii) generating a post-ablation surface topography by compounding theoretical 3D crater profiles (from i) or randomly selected experimental 3D crater profiles from the database (from ii), (iv) optimizing the ablation grid by minimizing the roughness of the post-ablation surface (from iii), and (v) simulating the output of the ICP-MS detector based on the optimized ablation grid (from iv) for virtual mapping of a phantom image. Several of these routines were incorporated into a generic model (not limited to LA–ICP–MS but applicable to all types of laser-based laser ablation devices) to simulate how the contraction of ablation grids may affect the post-ablation surface roughness and depth based on theoretical round and square super-Gaussian laser ablation crater profiles. The model is available as an online application (https://laicpms-apps.ki.si/webapps/home/).

## Results and Discussion

### LA Beam/Crater Profiles

To develop the geometrical modeling tool and get insights into the roughness of the post-ablation surface as a function of the ablation grid for specific crater profiles, we performed simulations with theoretical crater profiles generated by generic super-Gaussian beam profiles. These beam profiles can be described by the following equations for round (○) and square (□) beam profiles with beam size diameters BS(○) and BS(□), respectively
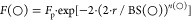
1and
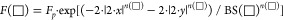
2where *F*(○) and *F*(□) are associated with the round and square fluence distribution in the ablation spot, respectively, with *r* and (*x*,*y*) denoting the radial distance and orthogonal coordinates, and *n*(○) and *n*(□) representing the order of the super-Gaussian probability density functions. The focused beam, defined as the distance from the center of the ablation spot to the edge where the fluence equals 1/*e*^2^ times the peak fluence *F*_p_, ablates the material above a certain threshold fluence *F*_th_.

**Figure 3:**
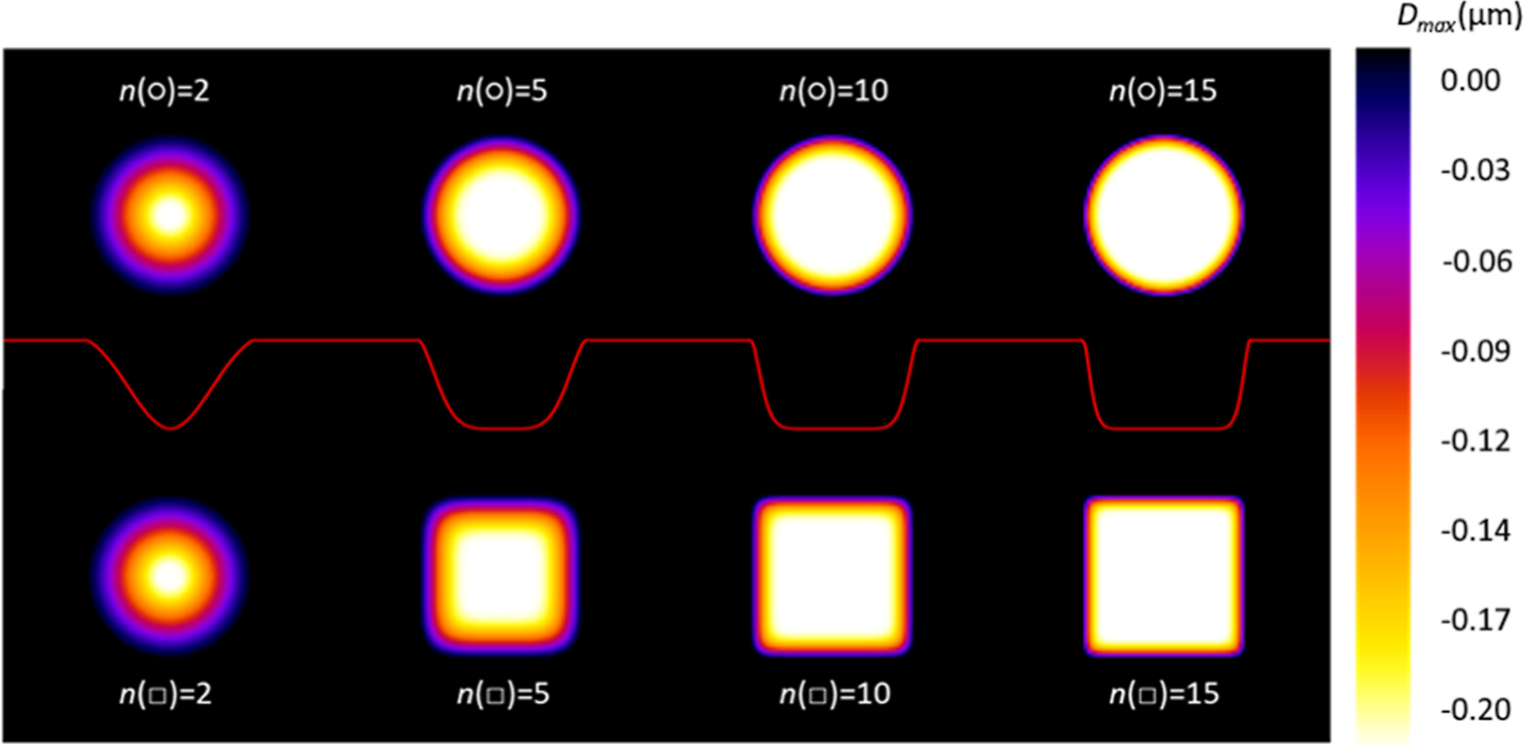
Theoretical crater geometry generated upon single shot laser ablation for similarly sized round (○) and square (□) beam profiles with super-Gaussian orders *n*(○) and *n*(□) of 2, 5, 10, and 15. The maximum ablation depth *D*_max_ is calculated via eq 3 based on real-life values for the spectral absorption coefficient α (0.0134 nm^–1^), threshold fluence *F*_th_ (0.263 J cm^–2^), and peak fluence *F*_P_ (4 J cm^–2^), all associated with ablation of borosilicate glass at 193 nm. The red trace shows that the linear depth profile through the center of the spots is identical for round and square spots.

The maximum ablation depth *D*_max_ per shot (in μm) increases logarithmically with the peak fluence *F*_p_ according to the following equation^14^
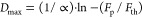
3where α is the spectral absorption coefficient. Figure 3 shows how the laser ablation spots, and the associated ablation craters, are directly linked to the super-Gaussian orders *n*(○) and *n*(□). The ablation craters produced by normal Gaussian beam profiles for round and square profiles are identical as eqs 1 and 2 converge to the same fluence distribution at *n*(○) = *n*(□) = 2. Analytical LA devices are preferably tuned to have a beam profile as close as possible to a flat-top beam profile, although for beam sizes <5 μm, the beam profile by definition approaches a normal Gaussian profile due to diffraction.

### Geometrical Modeling Fundamentals

Using round (○) or square (□) laser beams with diameters BS(○) or BS(□), respectively, the ablation grid coordinates (=ablation centers) are determined by the (horizontal and vertical) contraction factors *k*_*x*_(::) and *k*_*y*_(::) for the orthogonal grid and *k*_*x*_(∴) and *k*_*y*_(∴) for the hexagonal grid. The orthogonal ablation grid coordinates are given by
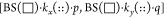
4where *p* and *q* are positive integers () associated with laser spot indexing in the ablation matrix. By definition, *k*_*x*_(::) = *k*_*y*_(::) = 1 for the closest packing of true square flat-top crater profiles (*n*(□) = ∞); for super-Gaussian crater profiles (*n*(□) < ∞), *k*_*x*_(::) = *k*_*y*_(::) < 1. The hexagonal ablation grid coordinates are given by
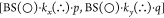
5aand
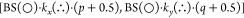
5b

for alternating grid rows *a* and *b*. By definition, for closest packing of true round flat-top crater profiles [*n*(○) = ∞], *k*_*x*_(∴) = 1 and *k*_*y*_(∴) = 0.5·√3 (see Figure 1); for super-Gaussian crater profiles [*n*(○) < ∞], *k*_*x*_(∴) < 1 and *k*_*y*_(∴) < 0.5·√3. Although most of the work focuses on ablation on symmetrical grid coordinates where *k*_*x*_(::) = *k*_*y*_(::) and *k*_*x*_(∴) = 2/√3·*k*_*y*_(∴), the online application (https://laicpms-apps.ki.si/webapps/home/) will also show how asymmetrical ablation grids may influence the post-ablation surface roughness and depth, whereas in the section on Surface Roughness and Ablation Depth Related to Image Quality for Actual Crater Profiles, this approach will be applied to the actual irregularly shaped experimental crater profiles given in Supporting Information-1. Minimizing the surface roughness through optimization of the contraction factors was carried out using the arithmetic mean roughness Ra (in μm)
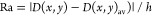
6where *D*(*x*,*y*) is the depth after ablation for each Cartesian coordinate (*x*,*y*) and *D*(*x*,*y*)_av_ denotes the average surface depth (=Σ*D*(*x*,*y*)/*h*) for *h* measurement points in the area ablated. The theoretical and experimental surface topographies are determined on a much finer grid than the ablation grids so that their roughness and depth can be accurately determined. Experimentally, ca. 33 points were measured in every μm^2^.

### Geometrical Modeling Results

**Figure 4:**
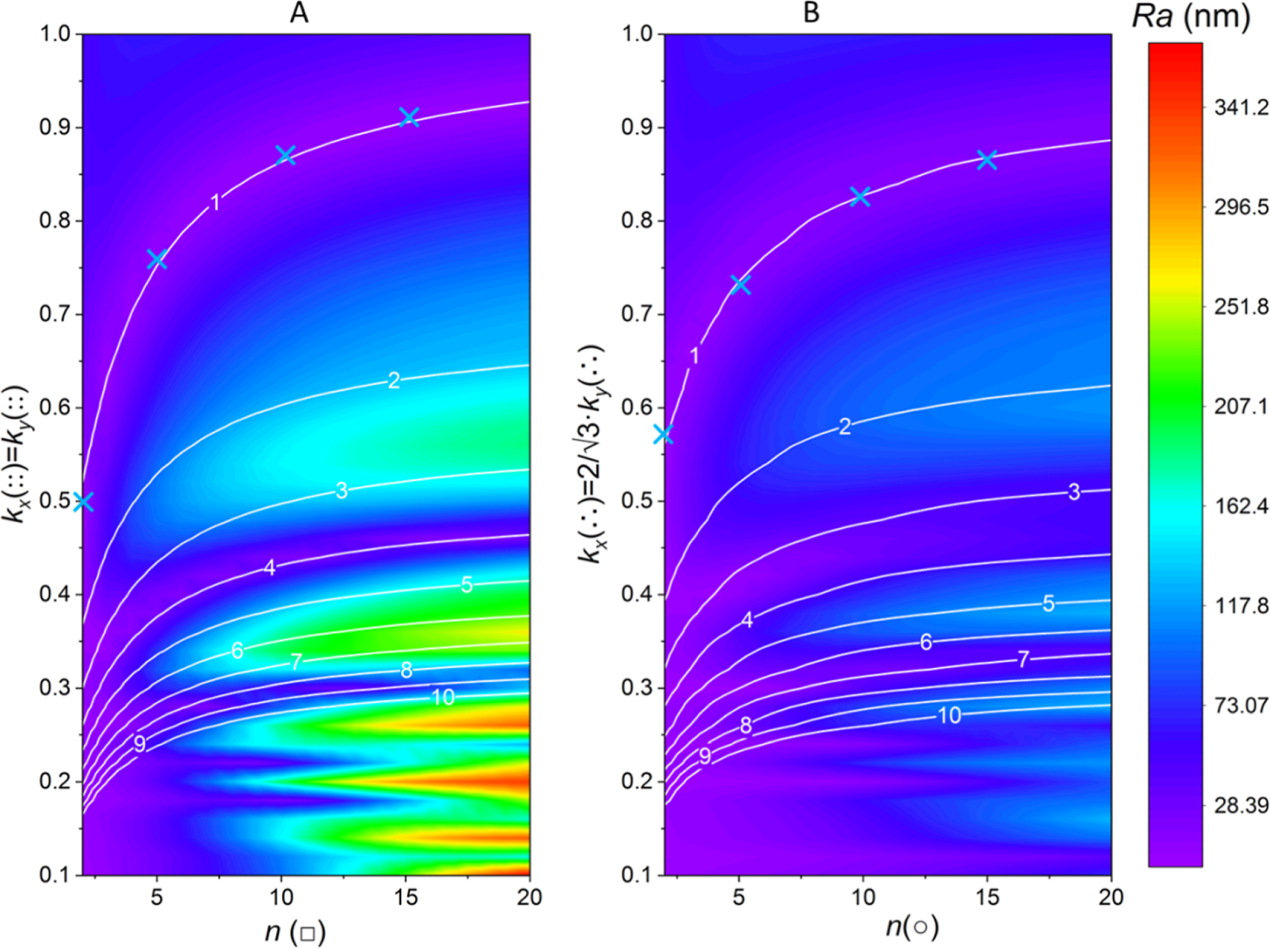
Optimizing the orthogonal (A) hexagonal (B) ablation grid contraction for theoretical square and round crater profiles *n*(□) and *n*(○) based on minimizing the surface roughness (Ra). The white lines are indicating the average ablation depth [*D*(*x*,*y*)_av_] normalized to the depth of a single laser ablation shot (*D*_max_). The light blue crosses indicate the optimal ablation grid contraction associated with the crater profiles given in Figure 3 for average layer depths of ca. one single laser shot.

By contracting the ablation grids in a symmetric manner, *i.e*., *k*_*x*_(::) = *k*_*y*_(::) and *k*_*x*_(∴) = 2/√3·*k*_*y*_(∴), for various crater profiles *n*(□) and *n*(○), insights can be gained into the post-ablation surface roughness and depth. Figure 4A,B demonstrates that symmetrical contraction of the grids results in multiple surface roughness minima, indicating that more than one smooth post-ablation layer can be generated as a result of various degrees of 2D spot overlapping. This leads to smooth post-ablation surface layers at several depths, as evident from the white lines with numbers which represent the average surface layer depth related to the maximum depth of a single laser shot. Both the orthogonal and hexagonal grids can be contracted to yield a smooth post-ablation surface for all crater profiles (*n*(□) and *n*(○) in the range 2–20) with a normalized average depth of one, although more contraction is necessary for lower super-Gaussian orders. Smooth, deeper ablation layers are associated with overlapping craters and may be responsible for blurring in LA–ICP–MS image maps.^8^ Nevertheless, for the orthogonal grid (Figure 4A), 2D overlapping of square spots still yields smooth depth layers at a normalized depth of approximately four for all crater profiles *n*(□), whereas deeper, smooth layers can only be generated at extreme overlapping of ablation craters with super-Gaussian orders less than 10. For the hexagonal grid (Figure 4B), the overall post-ablation surface smoothness seems less affected by pinpoint accuracy in the selection of the contraction parameters, as can be seen from the much broader purple regions compared to the orthogonal grid, and the fact that the overall depth range is narrower, as visible from the limited color range. As stated earlier, 2D overlapping of ablation spots by symmetrical contraction, leading to post-ablation layers much deeper than one shot, generates single pulse LA–ICP–MS maps with a higher pixel density, albeit with the risk of image blur.

**Figure 5:**
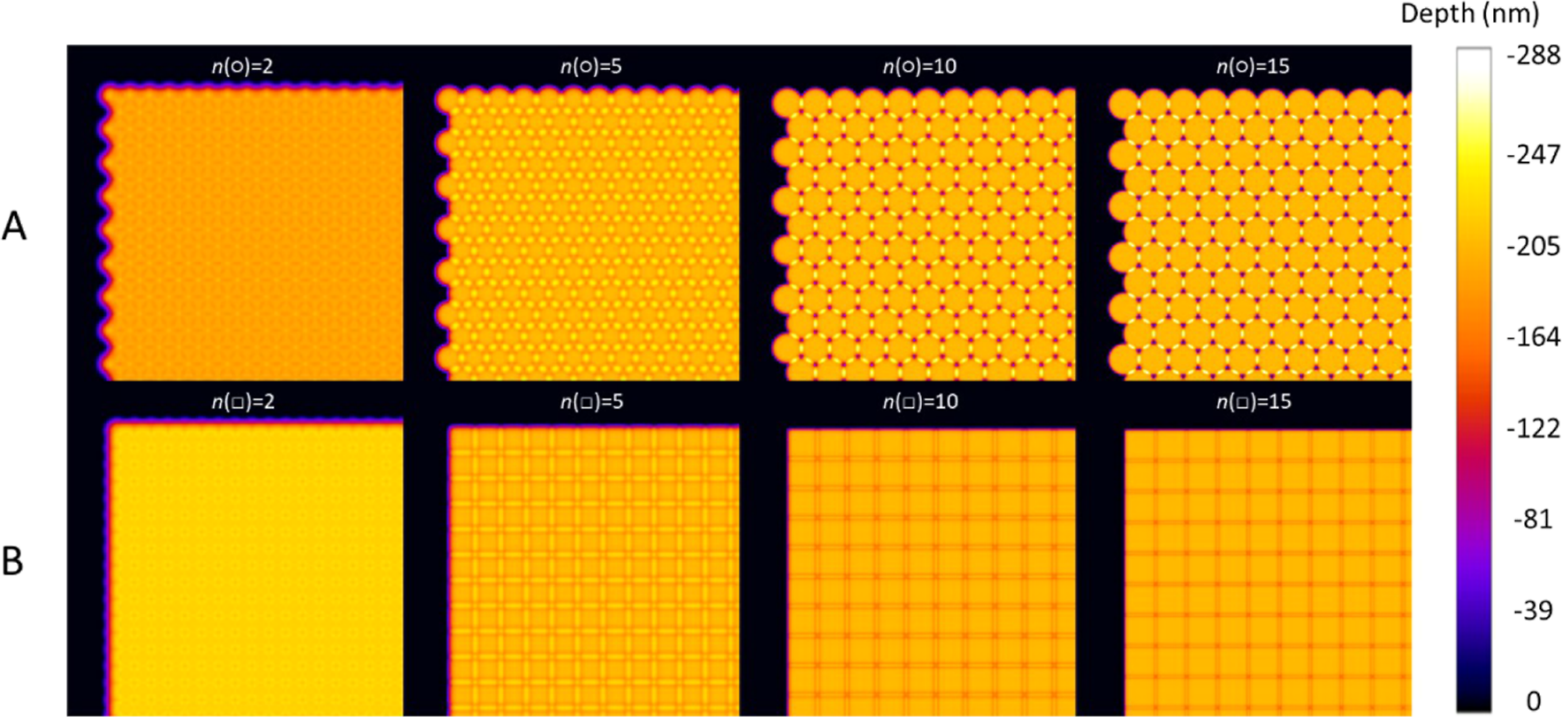
Simulation of post-ablation surface morphologies are shown for optimally contracted orthogonal (A) and hexagonal (B) laser ablation grids associated with the light blue crosses in Figure 4, for square and round crater profiles (with super-Gaussian orders of 2, 5, 10, and 15).

Figure 5 illustrates the simulated post-ablation surface morphologies associated with optimal contraction of the round and square ablation crater profiles, as indicated by the light blue crosses in Figure 4. Data associated with Figure 5 are presented in Table 1, which not only display the roughness of the post-ablation surfaces and their normalized layer depths but also the optimal contraction factors and relative spot densities in the layers. The table demonstrates that the theoretical surface roughness is generally better for square crater profiles compared to round ones, while the average ablation layer depths are similar, ranging from 93 to 108% of the maximum depth of a single laser shot (*D*_max_) for the respective beam profiles. As expected, the relative spot densities are highest for the Gaussian beam profiles on the orthogonal and hexagonal grids. For generic super-Gaussian beam profiles, the hexagonal grids can always be more densely packed than the orthogonal ones. Based on fitting of the averaged experimental crater profiles (Figure S2, Supporting Information SI-2), revealing super-Gaussian orders *n*(□) and *n*(○) of 8.979 and 7.006, respectively, we can assume that considerable gains in spot densities can be obtained.

**Table 1:** Characteristics of the Post-Ablation Surface Morphologies^a^

*n*(□)	*k*_*x*_(::)	*k*_*y*_(::)	Ra (nm)	*D*(*x*,*y*)_av_ (nm)	rel. spot density
2	0.5	0.5	2.5	219	4.00
5	0.76	0.76	7.5	199	1.73
10	0.88	0.88	8.0	196	1.29
15	0.92	0.92	7.3	197	1.18

*n*(○)	*k*_*x*_(∴) (μm)	*k*_*y*_(∴) [μm]	Ra [nm]	*D*(x,y)_av_ [nm]	rel. spot density
2	0.58	0.50	3.2	189	3.45
5	0.74	0.64	10.1	200	2.11
10	0.84	0.73	15.8	197	1.63
15	0.88	0.76	20.7	197	1.50

a The characteristics of the post-ablation surface morphologies in Figure 5 are presented for the square and round spots, including the horizontal and vertical grid spacings [*k*_*x*_(::), *k*_*y*_(∴), *k*_*x*_(::), and *k*_*y*_(∴)], roughness (Ra), average surface layer depth [*D*(*x*,*y*)_av_], and relative spot density, based on conditions associated with ablation of borosilicate glass at 193 nm (Figure 3). Spot densities are related to the density of a true flat-top square spot (*n*(□) = ∞) on an orthogonal ablation grid [*k*_*x*_(::) = *k*_*y*_(::) = 1].

So far, we only applied symmetrical contraction of ablation grids; however, a generic model, applicable for all kinds of laser-based ablation devices, incorporated in an online application (https://laicpms-apps.ki.si/webapps/home/), was developed to simulate how asymmetrical contraction of orthogonal (::) and hexagonal (∴) ablation grids affects the post-ablation surface roughness and depth as a function of theoretical square (□) or round (○) super-Gaussian laser ablation crater profiles, respectively (detailed information on the functioning of this app can be found in Supporting Information SI-3).

### Surface Roughness and Ablation Depth Related to Image Quality for Actual Crater Profiles

**Figure 6:**
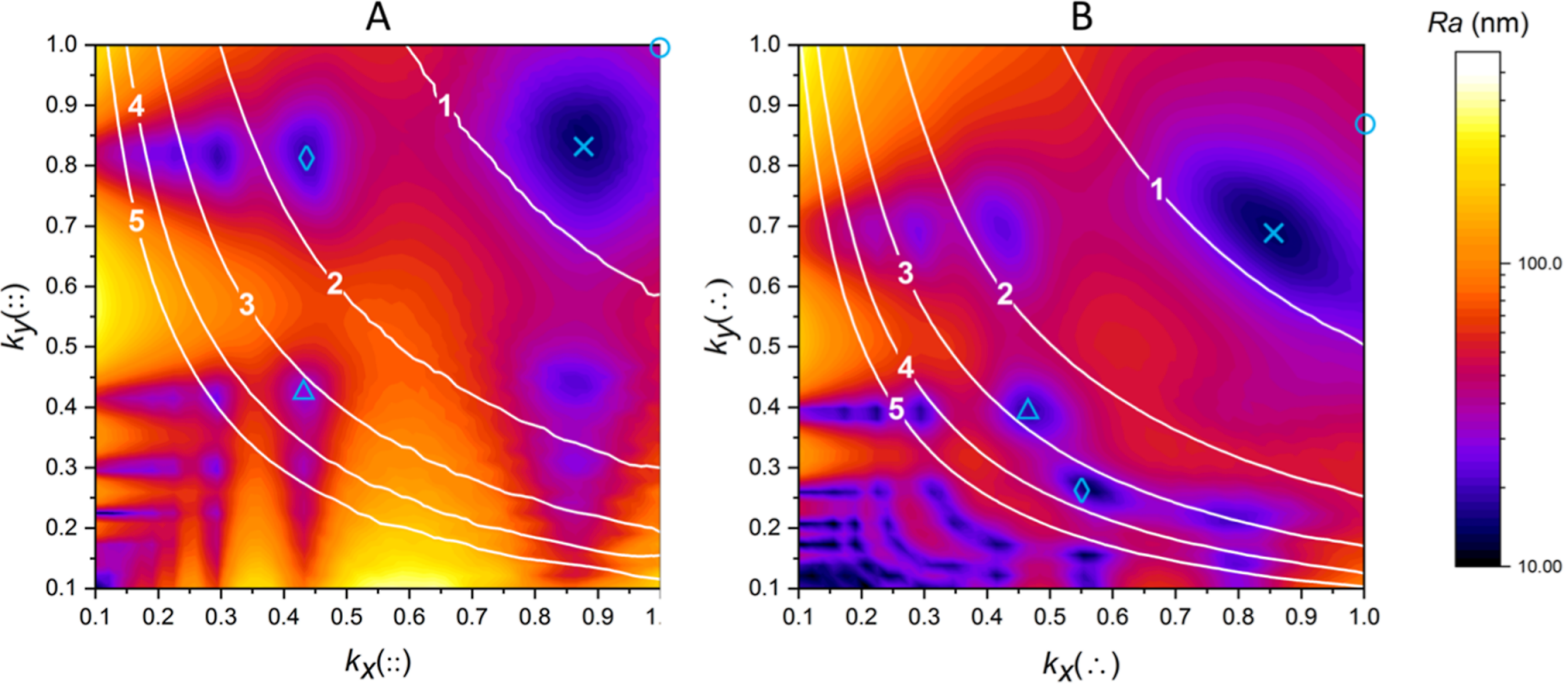
Optimization of orthogonal (A) and hexagonal (B) ablation grids based on contraction of the ablation grids for actual round and square 10 μm spots using the average of 50 experimental ablation craters (Figure S2, Supporting Information SI-2); colors are associated with surface roughness, and the white lines indicate the average surface layer depth related to the depth of a single shot. The light blue circles, crosses, and triangle denote different degrees of symmetric contraction, whereas the light blue diamonds denote an asymmetric contraction.

**Figure 7:**
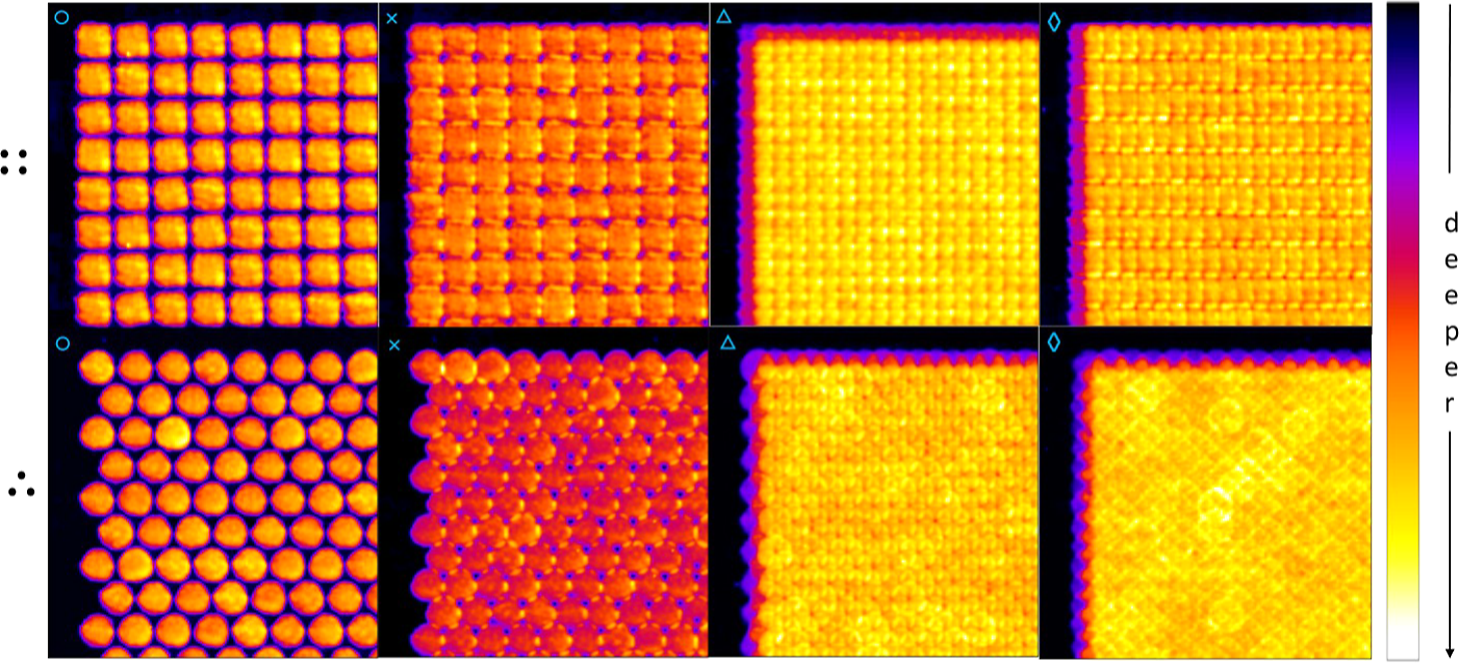
Simulated post-ablation surfaces based on randomly selected experimental crater profiles (Supporting Information-1) after laser sampling on orthogonal (::) and hexagonal (∴) grids associated with contraction factors indicated by light blue circles, crosses, triangles, and diamonds in Figure 6.

Orthogonal and hexagonal ablation grids were optimized for experimentally obtained 10 μm square and round crater profiles by independently adjusting the horizontal and vertical spacings. We used averaged experimental crater profiles and computationally compounded by placing them on ablation grids with contraction factors ranging from 0.2 to 1, in steps of 0.0173 (based on the step size of the optical profilometer). The contour plots in Figure 6 display the optimal parameters for ablation grid contraction that result in minimal roughness Ra and also show the average post-ablation layer depth normalized to the depth of a single laser shot. It can be seen that the predominant conditions for post-ablation smoothness are mostly associated with symmetric grid contraction (light blue crosses), leading to a post-ablation depth of roughly one shot at *k*_*x*_(::) = 0.86 and *k*_*y*_(::) = 0.86 for the orthogonal grid and *k*_*x*_(∴) = 0.83 and *k*_*y*_(∴) = 0.72 for the hexagonal grid. Indicated in Figure 6 are also the conditions where no contraction (light blue circles) and more extreme symmetric contraction (light blue triangles) and asymmetric contraction (light blue diamonds) have taken place. Based on these grid conditions, the post-ablation surface layers were simulated by random selection of experimental crater profiles shown in the videos in Supporting Information-1 and computationally compounding them by placing them on the ablation grids. Figure 7 shows the resulting simulated surface layer morphologies for the orthogonal (top) and hexagonal (bottom) grids, illustrating that contraction indeed delivers smoother post-ablation surfaces at different depth levels.

**Figure 8:**
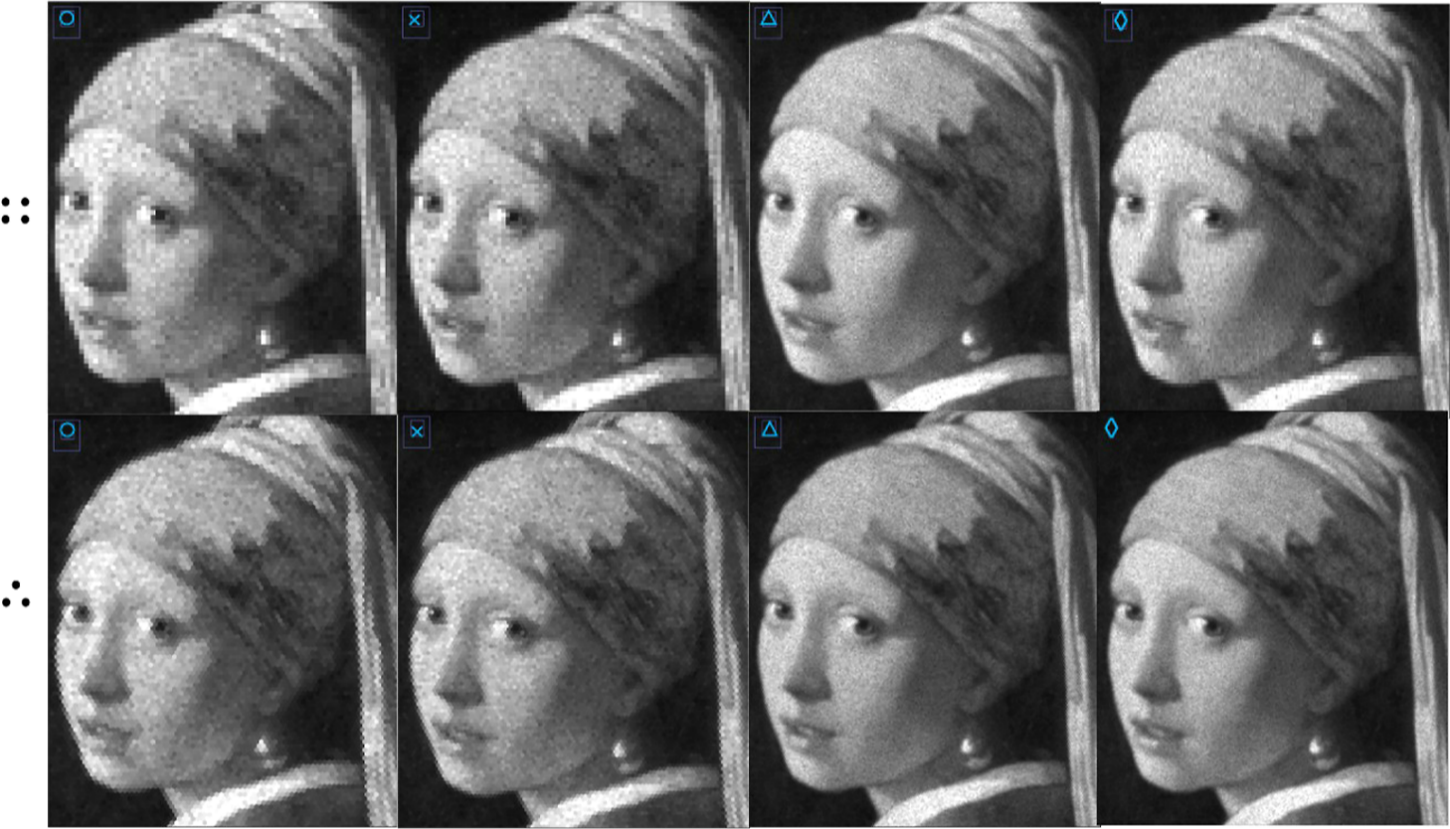
Simulated orthogonal (::) and hexagonal (∴) image maps after virtual ablation of a phantom image (Figure S4, SI-4), based on laser sampling associated with contraction factors indicated by light blue circles, crosses, triangles, and diamonds in Figure 6, and assuming a high concentration to minimize Poisson noise.

**Figure 9:**
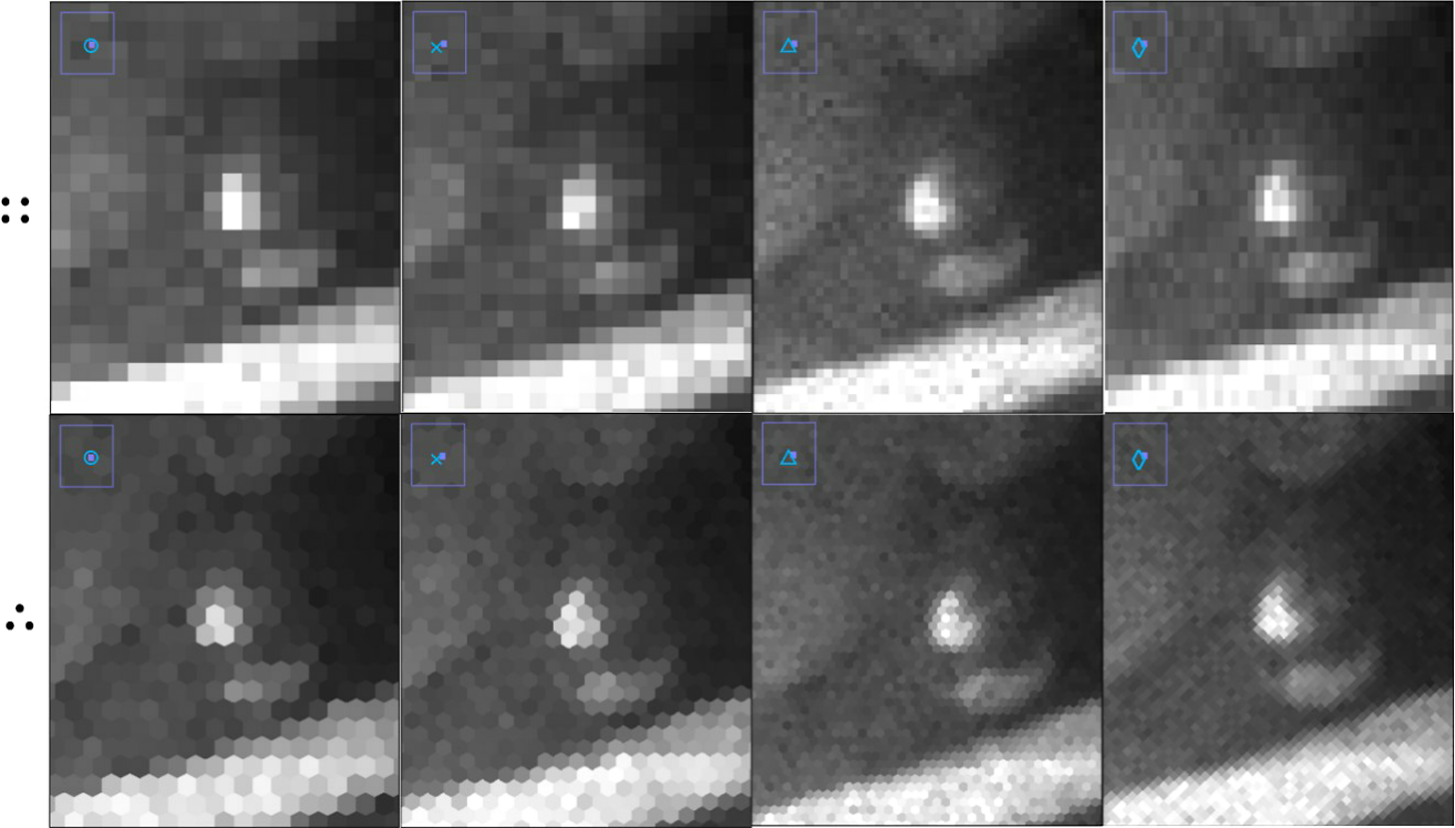
Simulated orthogonal (::) and hexagonal (∴) image maps of Figure 8 but now zoomed in on the earring.

In order to virtually ablate a phantom image (Figure S3, SI-4, “Girl with a Pearl Earring” by Johannes Vermeer, ca. 1665, from WikiMedia Commons), a 2D discrete convolution with a variable kernel was applied, again utilizing randomly selected experimental crater profiles from the databases in Supporting Information-1. It was hypothesized that laser sampling is the primary source of image noise when measuring high concentrations, resulting in minimal noise related to counting statistics. Results illustrated in Figure 8 suggest that ablation grid contraction improves image quality. In an upcoming study,^22^ it will be shown that increased pixel density not only leads to higher spatial resolution but also decreased image noise, though it is important to note that contraction should be moderate to avoid visible blurring. Additionally, Figure 9 demonstrates that zooming in on edge artifacts in hexagonal maps leads to improved perceived visual image quality. However, asymmetrical contraction of ablation grids results in distorted pixel shapes (rectangles and irregular hexagons), which may or may not improve image quality when targeting deeper surface layers in a single run. It may be more reliable to focus on mapping consecutive surface layers with optimized contraction factors. Nevertheless, this approach could be used to quickly expose a depth-controlled matrix layer with a smooth surface for subsequent LA–ICP–MS mapping.

## Conclusions

Single pulse LA–ICP–MS mapping typically employs spot-resolved surface analysis on an orthogonal grid, using square or round beam profiles to yield square pixels with dimensions equivalent to the laser beam size. However, as ablation craters are not typically cuboid or cylindrical in shape, and often have less steep edges, the ablated surface may exhibit a dimpled appearance. This indicates that the surface is not being accurately sampled, particularly for small round ablation spots (less than 10 μm) that exhibit a super-Gaussian crater profile and behave as objects with a “soft shell.” To improve the sampling efficiency, it is desirable to adapt the ablation grid and carefully oversample ablation spots in two dimensions. Specifically, arranging the ablation grid in a hexagonal pattern can increase pixel density by 15%, and further gains can be achieved by closer packing of “soft shell” spots to reduce the post-ablation surface roughness, depending on the crater profile.

This study has demonstrated that through the contraction of the ablation grid in single pulse LA–ICP–MS mapping, significant improvements can be made in post-ablation surface smoothness, spatial resolution, and control of the ablation layer depth. A geometrical modeling procedure, using actual input data related to 10 μm square and round ablation spots generated on a microscope glass slide, was used to simulate the post-ablation surface morphology. By carefully contracting the ablation grid and controlling 2D overlapping of ablation spots, it is possible to control the depth of ablation while still producing a smooth surface. For LA–ICP–MS mapping, it is recommended to use symmetrical contraction to moderate levels, such as a maximum of 2 × 2 crater overlaps, to prevent depth-related distribution differences and reduce the risk of blurring. Although blurring could be addressed through deconvolution, the relatively high noise in LA–ICP–MS mapping typically results in poor quality deconvolved maps.

It is important to note that for practical LA–ICP–MS mapping on contracted orthogonal and hexagonal ablation grids, stage inaccuracies are a concern, particularly for hexagonal mapping. In future research, this issue will be addressed through the development of piezoelectric stages in orthogonal and hexagonal arrangements, which will allow for the full benefits of ablation grid adaptation to be realized. Currently, an online application (https://laicpms-apps.ki.si/webapps/home/) is the closest available option for exploring the effects of ablation grid adaptation on post-ablation surface layer roughness and depth.

## Supporting Information Available

Explanation of laser ablation crater profile functions, surface roughness optimization web app user guide, and detailed description of virtual LA–ICP–MS mapping of a phantom image (PDF)
Individual laser ablation craters for round beam shape (AVI)
Individual laser ablation craters for square beam shape (AVI)

## Supplementary Material

ac3c00774_si_001.pdf

ac3c00774_si_002.avi

ac3c00774_si_003.avi
