# Supplementary material for: Exploring the Benefits of Ablation Grid Adaptation in 2D/3D Laser Ablation Inductively Coupled Plasma Mass Spectrometry Mapping through Geometrical Modeling
Source: Anal Chem. 2023 Jun 1;95(26):9863–71. doi: 10.1021/acs.analchem.3c00774 (PMC10323869; doi:10.1021/acs.analchem.3c00774)
Supplement: Supplementary file 1 — ac3c00774_si_001.pdf [file ac3c00774_si_001.pdf]

Supporting Information

Exploring the Benefits of Ablation Grid Adaptation in 2D/3D Laser Ablation Inductively Coupled Plasma Mass Spectrometry Mapping through Geometrical Modeling

Johannes T. van Elteren, Dino Metarapi*, Kristina Mervič and Martin Šala
Department of Analytical Chemistry, National Institute of Chemistry, Hajdrihova 19, SI-1000 Ljubljana, Slovenia.
*Email: dino.metarapi@ki.si

Table of Contents

SI-1. Experimental crater profiles for round and square beams 3

SI-2. Actual laser ablation crater profiles. 4

SI-2. Online Surface Roughness Optimization app incorporating the geometrical modeling tool. 5

SI-3. Virtual LA-ICP-MS mapping of a phantom image. 7

References 8

SI-1. Experimental crater profiles for round and square beams

The morphologies of the craters were measured by optical profilometry (Zegage PRO HR, Zygo Corporation, CT) and were used as input for the optimization of the orthogonal and hexagonal ablation grids through geometrical modeling. 3D information was recorded using a 50x magnification lens yielding a lateral resolution of 0.173 µm (equivalent to the step size) and surface topography repeatability better than 3.5 nm. The two videos (in avi format) show the individual topographic laser ablation crater profiles of 50 registered 10-µm round and square spots which are composed of roughly 2,600 and 3,300 optical profilometry measurement points, respectively.

SI-2. Actual laser ablation crater profiles.

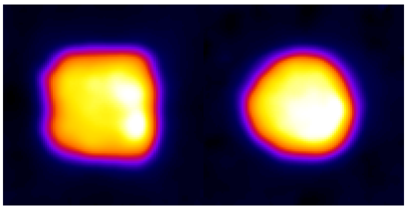

Figure S1. Average crater profiles based on the mean of the profiles given in the movie clips, and fitting them on the following functions to establish to super-Gaussian orders *n*(□) and *n*(○) (see Eqs. 1 and 2 in the main text for further details):

Square $A*\mathrm{exp}((-2*\mathrm{abs}(2*(x\text{-}x_0)^{n(\square)})\text{-}2*\mathrm{abs}(2*(y\text{-}y_0)^{n\square()}))/BS^{n(\square)})$

Round $A*\mathrm{exp}(-2*((2*\mathrm{sqrt}((x\text{-}x_0)^2 + (y\text{-}y_0)^2))/BS)^{n(\circ)})$

Fit Results:

Shape	*BS* (µm)	*n*	R^2
Square (□)	10	8.979	0.9847
Round (○)	10	7.006	0.9755

SI-3. Online Surface Roughness Optimization app incorporating the geometrical modeling tool.

A generic model (applicable for all kinds of laser-based ablation devices), incorporated in an online application (Surface Roughness Optimization app, https://laicpms-apps.ki.si/webapps/home/), was developed to simulate how variation of the ablation grid affects the post-ablation surface roughness and depth as a function of theoretical square (□) or round (○) super-Gaussian laser ablation crater profiles. The app allows for the optimization of laser ablation grid contraction to obtain a minimal post-ablation surface roughness.

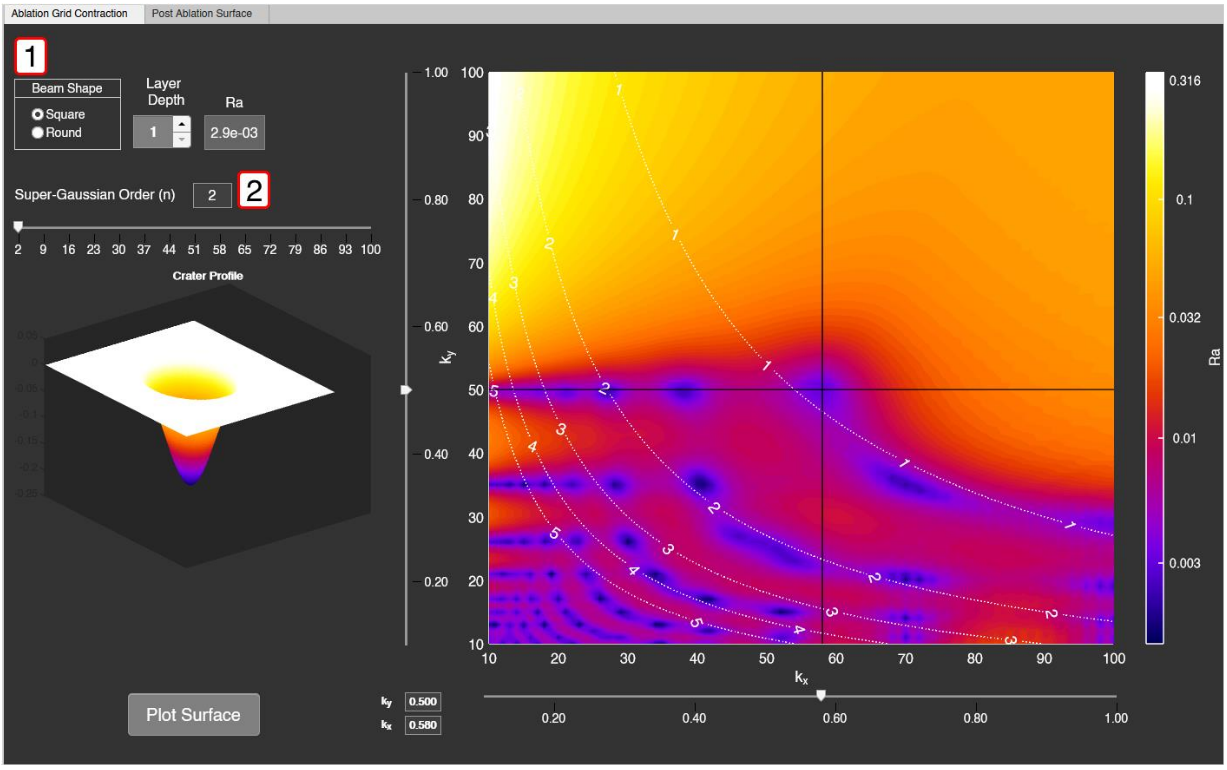

Figure S3a. The two primary inputs in the app are the beam shape (square or round) (**1**) and the super-Gaussian order (n) (**2**). For n values of 10 and above, the laser ablation crater profile progressively resembles a top hat profile. Selecting different values for n automatically updates the crater profile and the contour plot.

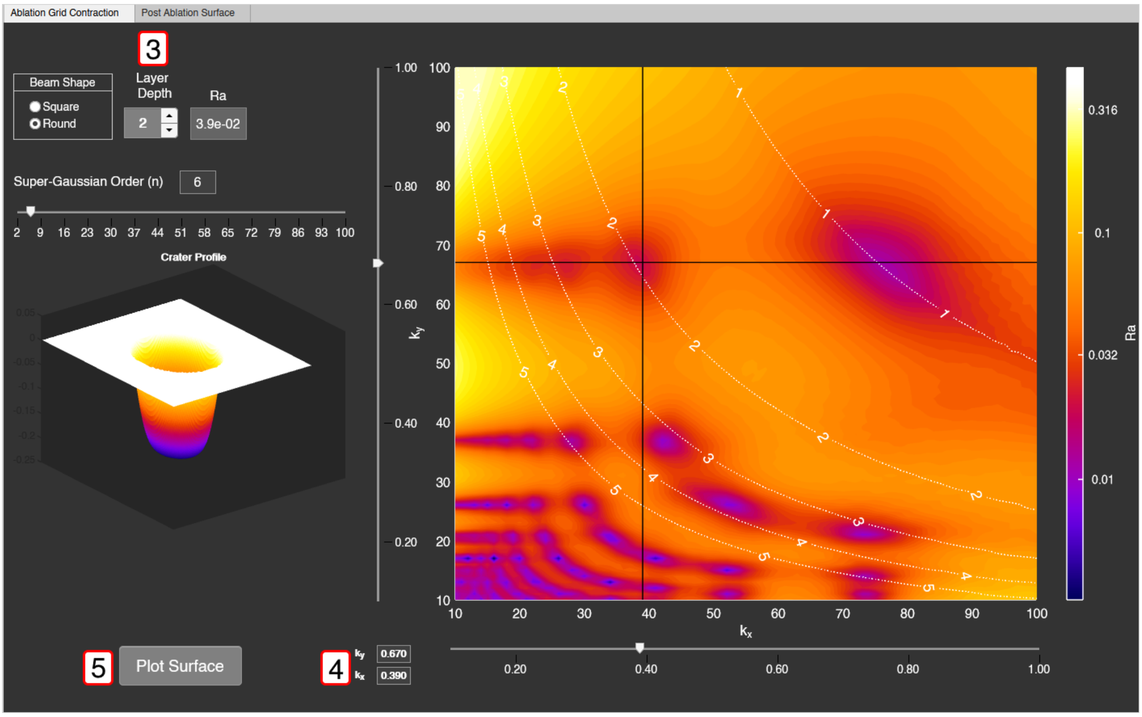

Figure S3b. By cycling through layer depths (**3**) it is possible to select the values for contraction factor k_x and k_y that result in post-ablation surfaces with the lowest roughness. The layer depth is normalized to the maximum depth of a single ablation crater. It is also possible to select custom values for the contraction factors, either by typing in the desired value (**4**) or using the sliders provided. Pressing the Plot Surface button (**5**) will use the currently selected k_x and k_y values for the selected crater profile to generate a virtual ablation surface topography.

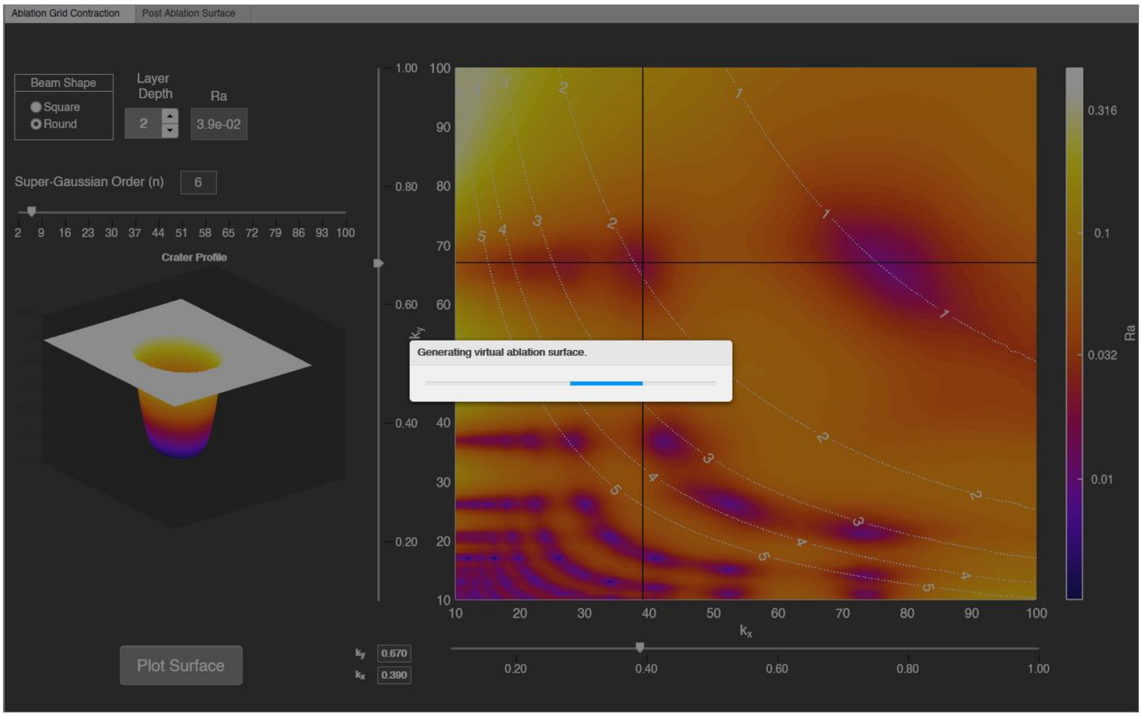

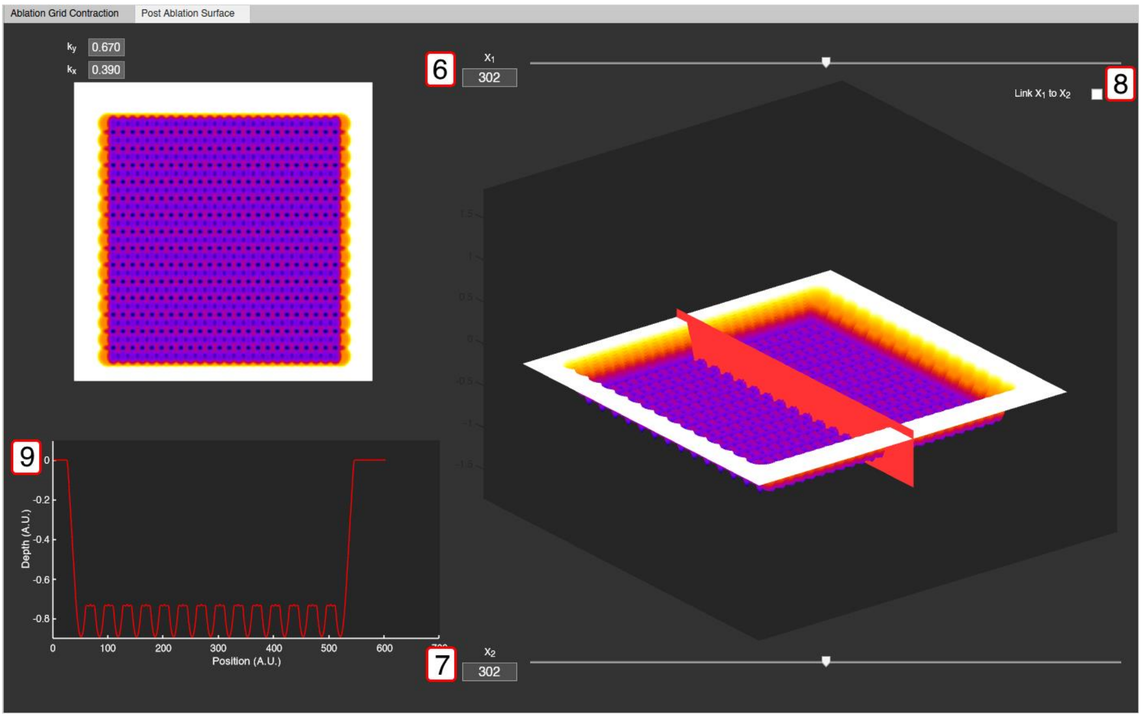

Figure S3c. After the computation is complete, the Post Ablation Surface tab will be activated, in which the user can observe the ablation surface topography. Using the X_1 and X_2 sliders (**6** and **7**) allows the user to display surface profiles at different positions. Clicking the "Link X_1 to X_2" checkbox (**8**) will lock the profile sampling to a straight line. The surface profile (expressed in arbitrary units) is displayed live in (**9**).

SI-4. Virtual LA-ICP-MS mapping of a phantom image.

To evaluate the image quality achievable with orthogonal and hexagonal ablation grid contraction, a phantom image (as shown in Figure S3) was subjected to virtual laser ablation inductively coupled plasma mass spectrometry (LA-ICP-MS) mapping. Numerical protocols,[1,2,3] based on 2D single pulse LA-ICP-MS mapping on orthogonal and hexagonal grids, were utilized to simulate the beam profile using discrete convolution with a variable kernel associated with random selection of experimental crater profiles from the databases in ESI-1. Special considerations are necessary when processing virtual LA-ICP-MS data from hexagonal ablation grids and constructing associated element maps. The data must be positionally aligned into a staggered/interleaved formation associated with the hexagonal ablation grid. This requires sufficient upscaling of the data matrix prior to hexagonal rearrangement and calculation of Voronoi tessellations using the voronoin function in MatLab. More details on virtual mapping on hexagonal ablation grids and subsequent data processing will be discussed in an upcoming paper.[4]

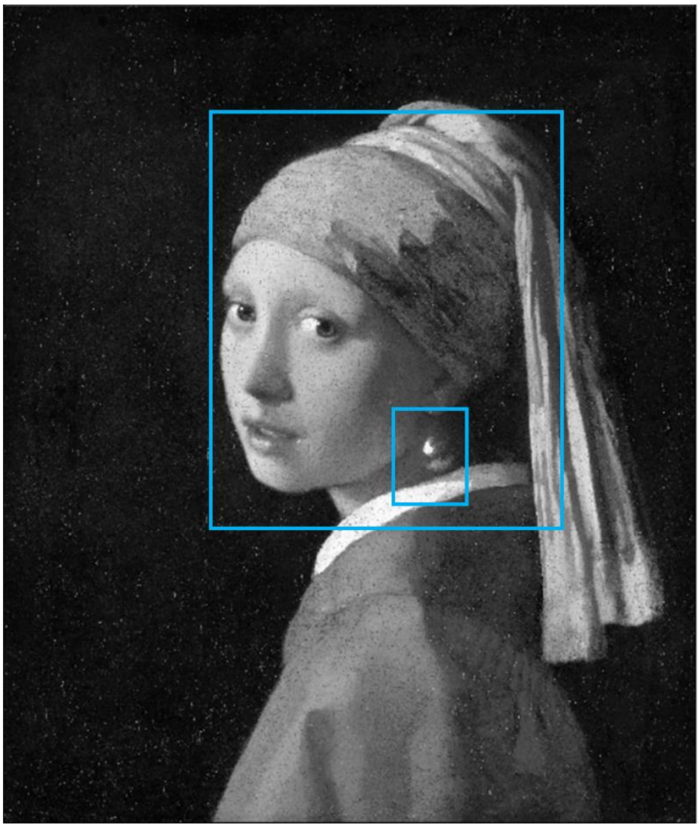

Figure S4. The painting "Girl with a Pearl Earring" by Johannes Vermeer, created in 1665, was obtained from WikiMedia Commons and converted into an 8-bit grayscale image with a resolution of 8,443x10,000 pixels and assumed pixel dimensions of 0.173x0.173 μm^2. The blue frames in the image indicate the areas shown in Figures 8 and 9.

References

1. Van Elteren, J. T.; Šala, M.; Metarapi, D. Comparison of Single Pulse, Multiple Dosage, and 2D Oversampling / Deconvolution LA-ICPMS Strategies for Mapping of (Ultra)Low-Concentration Samples. Talanta 2021, 235 (August). https://doi.org/10.1016/j.talanta.2021.122785.
2. Van Elteren, J. T.; Šala, M.; Šelih, V. S. Perceptual Image Quality Metrics Concept in Continuous Scanning 2D LA-ICP-MS Bioimaging. Anal. Chem. 2018, 90, 5916–5922. https://doi.org/10.1021/acs.analchem.8b00751.
3. Van Elteren, J. T.; Izmer, A.; Šelih, V. S.; Vanhaecke, F. Novel Image Metrics for Retrieval of the Lateral Resolution in Line Scan-Based 2D Imaging via an Experimental-Modeling Approach. *Anal. Chem.* **2016**, *88*, 7413–7420. https://doi.org/10.1021/acs.analchem.6b02052.
4. Metarapi, D.; van Elteren, J.T. High-Resolution Single Pulse LA-ICP-MS Mapping via 2D Sub-Pixel Oversampling on Orthogonal and Hexagonal Ablation Grids – a Computational Assessment. *IEEE Trans. Image Process.*, submitted.
